# Analytical Low-Dose CBCT Reconstruction Using Non-local Total Variation Regularization for Image Guided Radiation Therapy

**DOI:** 10.3389/fonc.2020.00242

**Published:** 2020-02-27

**Authors:** James J. Sohn, Changsoo Kim, Dong Hyun Kim, Seu-Ran Lee, Jun Zhou, Xiaofeng Yang, Tian Liu

**Affiliations:** ^1^Department of Radiation Oncology, Emory University, Atlanta, Georgia; ^2^Department of Radiological Science, The Catholic University of Pusan, Busan, South Korea; ^3^Department of Biomedical Engineering, The Catholic University of Korea, Seoul, South Korea

**Keywords:** low-dose CBCT, non-local total variation, compressed sensing, image reconstruction, image-guided radiation therapy (IGRT)

## Abstract

**Purpose:** Conventional iterative low-dose CBCT reconstruction techniques are slow and tend to over-smooth edges through uniform weighting of the image penalty gradient. In this study, we present a non-iterative analytical low-dose CBCT reconstruction technique by restoring the noisy low-dose CBCT projection with the non-local total variation (NLTV) method.

**Methods:** We modeled the low-dose CBCT reconstruction as recovering high quality, high-dose CBCT x-ray projections (100 kVp, 1.6 mAs) from low-dose, noisy CBCT x-ray projections (100 kVp, 0.1 mAs). The restoration of CBCT projections was performed using the NLTV regularization method. In NLTV, the x-ray image is optimized by minimizing an energy function that penalizes gray-level difference between pair of pixels between noisy x-ray projection and denoising x-ray projection. After the noisy projection is restored by NLTV regularization, the standard FDK method was applied to generate the final reconstruction output.

**Results:** Significant noise reduction was achieved comparing to original, noisy inputs while maintaining the image quality comparable to the high-dose CBCT projections. The experimental validations show the proposed NLTV algorithm can robustly restore the noise level of x-ray projection images while significantly improving the overall image quality. The improvement in normalized mean square error (NMSE) and peak signal-to-noise ratio (PSNR) measured from the non-local total variation-gradient projection (NLTV-GPSR) algorithm is noticeable compared to that of uncorrected low-dose CBCT images. Moreover, the difference of CNRs from the gains from the proposed algorithm is noticeable and comparable to high-dose CBCT.

**Conclusion:** The proposed method successfully restores noise degraded, low-dose CBCT projections to high-dose projection quality. Such an outcome is a considerable improvement to the reconstruction result compared to the FDK-based method. In addition, a significant reduction in reconstruction time makes the proposed algorithm more attractive. This demonstrates the potential use of the proposed algorithm for clinical practice in radiotherapy.

## Introduction

CBCT has been widely adopted for radiotherapy for tumor visualization and localization (1, 2). However, CBCT delivers considerable imaging dose to the patient through ionizing x-rays. The cumulative imaging dose from repeated CBCT scans is clinically significant, and the optimization of x-ray exposure conditions is necessary to meet dose constraints. Moreover, keeping the low dose in CBCT with as low as reasonably achievable (ALARA) principles, it is desirable to reduce imaging dose (3–5). Therefore, minimizing imaging dose while maintaining adequate image quality for accurate tumor visualization is highly desirable in the clinical setting.

Imaging dose is proportional to the exposure level (mAs) from the x-ray tube of the CBCT imaging system. Reducing the exposure level reduces the fluence to x-rays projected onto the patient and thus reduces the CBCT imaging dose. However, an excessive reduction in exposure amplifies the noise level of the projection image due to the photon starvation effect. Moreover, when using the conventional Feldkamp, Davis, and Kress (FDK) algorithm for reconstruction, it is difficult to sustain the image quality due to the amplification of noise with the high bandpass filter applied to noisy projection data (6–8).

To solve this problem, iterative reconstruction methods based on total variation (TV) have been proposed to enhance the image quality of low-dose CT/CBCT. These methods effectively reduce the overall noise; however, TV normalization attempts to isotropically over smooth the image by applying the penalty gradient to detail across the entire image. In order to improve such limitations of conventional TV-based algorithms, the Edge-based TV (EPTV) and adaptive weighted TV (awTV) methods have been developed. EPTV and awTV calculate isotropic as well as anisotropic details computed by the exponential-type operator of the image slope from the intermediate image generated during iterations (9–11). In these recalibration-based models, the value of TV penalties is suppressed in the edge component where the image gradients are high to reduce edge smoothing. Although improvements were made compared to conventional TV-based methods, the ability to preserve detail (e.g., edges) in the conventional TV-based approaches were constrained by the intermediate image quality during the iterative process, which depends on the level of noise per projection. As a result, at very low mAs conditions, the edges are still blurred.

Another challenge of TV-based CBCT reconstruction is long reconstruction times (3–8, 12). During the iterative process of solving the TV-based least squares problem, multiple forward, and backward projections of large datasets must be calculated in each iterative process. This computation process is widely known to be computationally heavy and long to process. To make a CBCT reconstruction process practical for its clinical use, reconstruction must be completed within a few minutes (clinically feasible timeframe).

In this study, we propose a non-iterative analytical image reconstruction algorithm based on the recovery of noisy CBCT projections. A new edge-preserving denoising method called the non-local total variation model is applied to restore the signal to noise ratio (SNR) of noisy, low-dose CBCT projections to a quality that is comparable to high dose CBCT projection images. A comprehensive evaluation of our approach applied to images from a numerical phantom, as well as a physical phantom, is presented.

## Method and Materials

Recently, non-local total variation (NLTV) has drawn much attention to the image denoising problem. Based on the advantages of non-local means (NLM), Gilboa and Osher introduced a non-local variational model to improve texture further (13).

Given an x-ray projection domain P (P can be sub-domain of ℝ^2^), consider a noisy CBCT projection image **I** as a mapping of P → ℝ, where **I** is obtained from a perfectly denoised unknown image *u*. In this form, the proposed mathematical formulation of NLTV based denoising on the CBCT projection data can be written as

(1) $$\begin{array}{r} \underset{u\in \Omega }{\text{argmin}}\lambda J_{NLTV}\left(u\right)+∥u-\text{I}{∥}^{2} \end{array}$$

where Ω is either ℝ^P^ (in a discrete level) or the space of the functions of bounded variation and *J*_*NLTV*_ (u) is defined by

(2) $$\begin{array}{r} \int_{P}\sqrt{\int_{B}{\left(u\left(p\right)-u\left(p+q\right)\right)}^{2}v\left(p\right)dqdp} \end{array}$$

where *B* is a search window, and the weights *v* are defined as

(3) $$\begin{array}{r} v\left(p\right)=exp\left(-\frac{\sum_{k=-B/2}^{B/2}G\left(k\right)\cdot |u\left(p+k\right)-u\left(p-k\right){|}^{2}}{{2h}_{0}^{2}}\right) \end{array}$$

Here, *G* is a Gaussian kernel with patch size *B* and filtering parameter *h*_0_. Unlike conventional uniform TV, it assigns non-uniform weights to the global search area such that the smaller the difference for the pair of image intensities, the greater weight is imposed. In this work, the patch of the Gaussian kernel is defined to be 10 × 10 with unit variance. The search area for NLTV to seek a similar block is set to be 300 × 300 with unit variance, and the filtering parameter was set to be between 1 and 6 times of the image background. The similarity is evaluated by a given threshold α where the criterion by which two blocks can be considered similar is defined as

(4) $$\begin{array}{r} ∥u_{i}-u_{j}{∥}^{2}\leq \alpha \end{array}$$

If the similarity is greater than the threshold, it is considered dissimilar and ignored, and only the similar blocks are used in the NLTV calculation. To reduce the number of blocks considered in the searching and accelerate the speed of computation, a discriminator method is used. The basic idea is that considering the two estimated blocks are similar, they should have similar average intensities (pixel values) and standard deviations. In this manner, the series of block means and standard deviations can be computed in advance. If the blocks are considered similar, the distance will be calculated. Otherwise, if the blocks are considered dissimilar, then the distance will be set to ∞.

(5) $$\begin{array}{l} d\left(u_{i},u_{j}\right)=\{\begin{array}{cc} \frac{∥u_{i}-u_{j}{∥}^{2}}{B^{2}} & if\;mean\left(u_{i}-u_{j}\right)\leq \lambda_{1}\cap var\left(u_{i}-u_{j}\right)\leq \lambda_{2}\\ \;\infty & \;Otherwise \end{array} \end{array}$$

**Figure 1:**
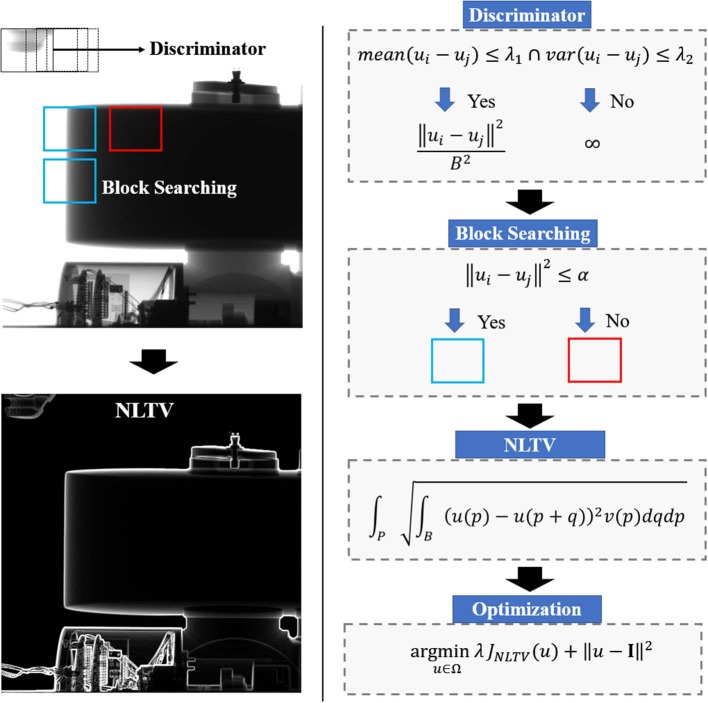
Work flow design of proposed NLTV filtering process. NLTV comprises of four steps: discriminator, block searching, NLTV calculation and optimization.

In this manner, the maps of local means and standard deviations are computed in advance, and repetitive calculation of the search window can be prevented. Figure 1 shows the workflow of the NLTV denoising workflow at a given projection.

**Figure 2:**
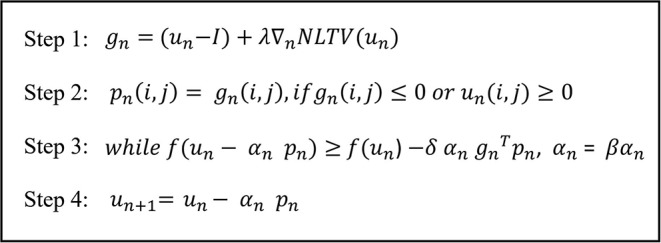
Illustration of the computational process required at each iteration for the GPSR-NLTV algorithm.

After NLTV is computed, the final step is to solve the optimization problem defined by Equation (1). Here, the gradient projection for sparse reconstruction (GPSR) was implemented that searches the global minimum of convex function with the projected gradient at each iteration. It is started by computing the gradient of the energy function consisting of the data fidelity and the NLTV penalty terms, followed by projecting in the direction of the gradient. Next, a back-tracking line-search is conducted by evaluating the energy function with a decreasing step-size α_n_ until the Armijo condition is satisfied (14). The latest commercialized algorithm is fast since it works with the iterations ten times only; however, there is no convergence guarantee. It means that because it takes too long to perform the forward and backward projection so that it is performed ten times only, taking into account the appropriate throughput time, and stopping the iteration. On the other hand, our algorithm processes directly from the projection data, so it does not need forward and backward projection, so it is more efficient. This not only guarantees a monotonic decrease in the objective function but also satisfies a sufficient decrease criterion for convergence to the optimal solution. Figure 2 illustrates the GPSR optimization process at each iteration.

**Figure 3:**
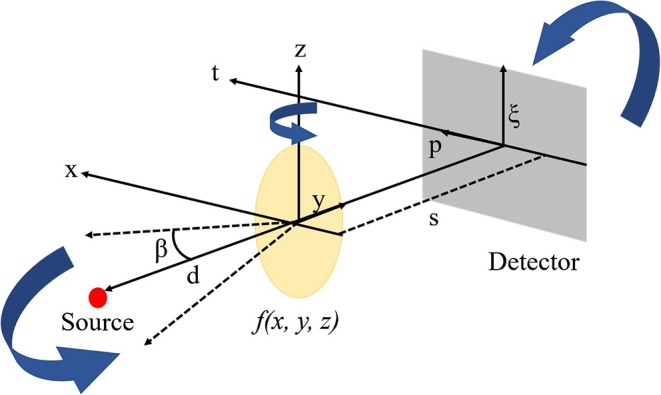
Reconstruction geometry of CBCT.

After the noisy low-dose projections are restored using the GPSR-NLTV method, an analytical reconstruction technique was used to generate results. In this study, we used the standard FDK algorithm due to its simplicity and efficiency. Briefly, the axial voxel information at position (x,y,z), denoted by f(x,y,z), can be calculated from the following equation:

(6) $$\begin{array}{r} \text{f}(\text{x},\text{y},\text{z})\;=\;\frac{1}{N_{0}}\int_{\theta =0}^{360}\int_{-\infty }^{\infty }\frac{d}{\sqrt{d^{2}+p^{2}+\xi^{2}}}R\left(\beta ,p,\xi \right)h\left(\frac{d\cdot t}{d-s}-p\right)dpd\beta \end{array}$$

where *N*_0_ refers to the total number of projections, β, d, p, and ξ refer to the angle of each projection, source-to detector distance, detector axis perpendicular, and parallel to the axis of rotation, respectively, R(·) refers to restored CBCT projections, and h(·) refers to the convolutional high pass filter. Figure 3 shows the corresponding reconstruction geometry.

We used two phantoms in this study: a cylindrical Catphan 600 phantom and a physical anthropomorphic head phantom. The projections were acquired using the x-ray Volumetric Imaging system (XVI™) integrated with an Elekta Versa HD Unit (Elekta Oncology Systems Ltd, Crawley, UK). The number of CBCT projections for a full 360° rotation scan was 670. The physical dimensions of each acquired x-ray projections were 410 × 410 mm^2^, containing 1,024 × 1,024 pixels. For each phantom, the x-ray tube current was configured at 0.1 mA (low-dose) and 1.6 mA (high-dose) during CBCT data acquisitions. In both phantom simulations, the tube voltage was configured at 100 kVp, and the period of the x-ray pulse in each projection scan was set at 10 ms. The projection data were acquired in full-fan mode with a bowtie filter. The source-to-isocenter distance was 1,000 mm, and the source-to-detector distance was 1,500 mm.

## Results

**Figure 4:**
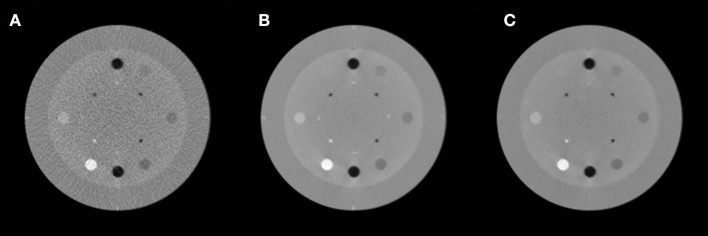
The images of the Catphan 600 phantom reconstructed using noisy, low-dose projection **(A)**, high-dose projection **(B)**, and proposed method **(C)**.

**Figure 5:**
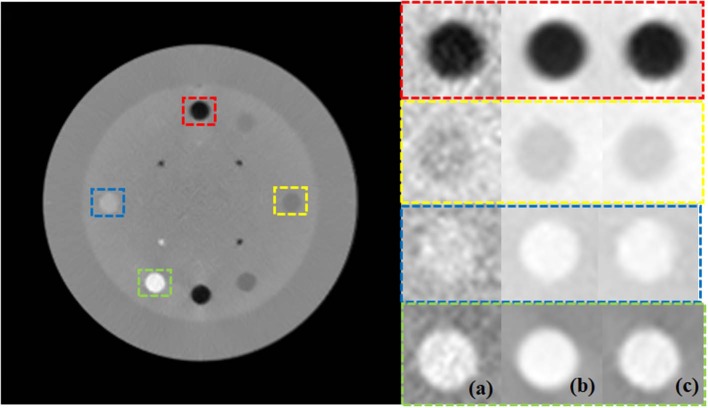
Zoomed images of ROIs of the contrast of Catphan 600 phantom using noisy low-dose projection **(A)**, high-dose projection **(B)**, and proposed method **(C)**.

**Figure 6:**
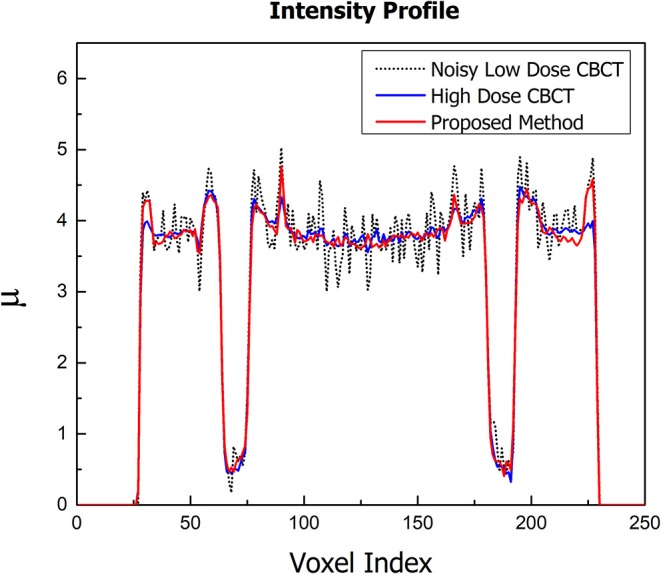
Horizontal profiles through the center of the reconstructed images of Catphan 600 phantom.

The images of the Catphan 600 phantom reconstructed using noisy, low-dose projections, high-dose projections, and the proposed method are shown in Figure 4. Figure 4A displays the CBCT images reconstructed with a conventional FDK method with a ramp high pass filter using the low-dose projection data. In the figure, it is evident that severe noise and artifacts are noticeable. Figure 4C shows the proposed low-dose CBCT reconstruction using NLTV-GPSR from the low-dose projection data. It is noticed that the noise, as well as the artifacts, are significantly reduced, and quality comparable to that of the CBCT reconstructed from high-dose projections is achieved. To further compare the performance of our proposed NLTV-GPSR algorithm, a magnified view of ROIs of the contrast of Catphan 600 phantom is shown in Figure 5. It is clear that the proposed NLTV-GPSR algorithm achieves better performance compared to the original low-dose CBCT images in terms of both artifact removal and preserves more sharper edges. Figure 6 draws horizontal profiles through the center of the reconstructed CBCT in Figure 4. It is shown that the proposed NLTV-GPSR algorithm achieves a closer profile compared with the high-dose reference, especially on the edge of the contrast region. This profile analysis further reveals the higher low-contrast detectability and detail-preserving performance of the proposed NLTV-GPSR algorithm compared to conventional approaches.

Mathematically, PSNR, and NMSE are defined as:

(7) $$\begin{array}{r} \text{PSNR }=\;10\log\left(\frac{{\max\left\{\mu_{high\;dose}\right\}}^{2}}{∥{\mu \;-\;\mu }_{high\;dose}{∥}^{2}/K}\right) \end{array}$$

(8) $$\begin{array}{r} \text{NMSE }=\;\frac{∥{\mu \;-\;\mu }_{high\;dose}{∥}^{2}}{∥{\;\mu }_{high\;dose}{∥}^{2}} \end{array}$$

where μ represents the intensity value of the test CBCT image, μ_highdose_ represents the intensity value of the reference high-dose CBCT, and *K* refers to the total number of CBCT voxels. Table 1 lists the measured PSNR and NMSE values of the reconstructed CBCTs, comparing the original low-dose CBCT (LD-CBCT) and high-dose CBCT (HD-CBCT) and the proposed method. The improvement of the proposed NLTV-GPSR algorithm is clear in comparison with the uncorrected low-dose CBCT images. This shows that the proposed NLTV-GPSR algorithm can achieve better noise suppression compared to uncorrected low-dose CBCT images.

**Table 1:** The PSNR and NMSE values of the reconstructed CBCTs comparing with original low-dose CBCT (LD-CBCT), and high-dose CBCT (HD-CBCT), and the proposed method (NLTV-GPSR) as reference.

**Methods**	**LD-CBCT**	**HD-CBCT**	**NLTV-GPSR**
PSNR (dB)	25.28	41.07	39.53
NMSE (1e-3)	16.27	1	1.298

In addition to comparing PSNR and NMSE, the two ROIs were selected as indicated by the green and blue squares (ROI 1, ROI 2) in Figure 5 and compared the contrast-to-noise ratio (CNR) at each ROI. The CNR in this study is defined as:

(9) $$\begin{array}{l} \text{CNR}=\frac{\left|\mu_{ROI}-\mu_{BG}\right|}{\sqrt{\sigma_{ROI}^{2}+\sigma_{BG}^{2}}} \end{array}$$

where μ_ROI_ refer to the average value of the voxels inside the ROI, μ_BG_ refer to the average value of the voxels of the CBCT background, and σ_ROI_, σ_BG_ refer to standard deviation of the voxel values at each ROI and the CBCT background, respectively. Here, the size of ROIs was set as 20 × 20. Table 2 displays the measured CNRs of the reconstructed CBCT images using four different reconstruction methods. The differences of CNRs from the high-contrast region of ROI 1 confirm the gains from the proposed algorithm, and the quality is comparable to high-dose CBCT.

**Table 2:** Measured CNRs of the reconstructed CBCT comparing with original low-dose CBCT (LD-CBCT) and high-dose CBCT (HD-CBCT) and the proposed method (NLTV-GPSR).

**Methods**	**LD-CBCT**	**HD-CBCT**	**NLTV-GPSR**
CNR (ROI 1)	2.237	3.208	3.187
CNR (ROI 2)	0.332	1.363	1.227

**Figure 7:**
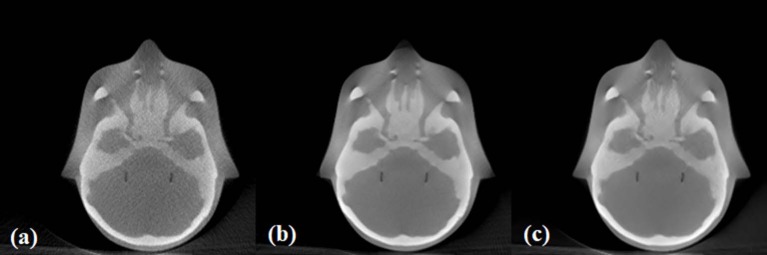
The images of the anthropomorphic head phantom reconstructed using noisy, low-dose projections **(A)**, high-dose projections **(B)**, and the proposed method **(C)**.

The images of the anthropomorphic head phantom reconstructed using noisy, low-dose projections, high-dose projections, and the proposed method are shown in Figure 7. Specifically, Figure 7A displays reconstructed low-dose CBCT image from the noisy, low-dose projections, Figure 7B displays the proposed low-dose CBCT reconstructed using the NLTV-GPSR algorithm, and Figure 7C displays the reference high-dose CBCT. It is clear that noise and artifacts are significantly reduced while the detailed edge profile is preserved when using the NLTV-GPSR algorithm (Figure 6B). In correspondence with the Catphan phantom's result, this confirms that the proposed NLTV-GPSR can achieve better image quality compared to the original low-dose CBCT in terms of both edge preservation as well as the artifact suppression.

## Discussion

Implementing a low-dose CBCT reconstruction algorithm into a clinical setting is still challenging since most of the proposed solutions to this problem are iterative rather than analytical. All iterative methods involve at least a single back- and forward-projection, in addition to correcting noise penalties in the reconstruction domain. Recent studies have focused on achieving faster convergence on the convex optimizer; however, at least 20 iterations are required in order to achieve clinically usable image quality (15–17). Although a significant amount of computational time can be reduced by parallelizing the process of forward- and back-projection operation using a Graphical Processing Unit (GPU), significant (>80%) amount of the time is still spent on calculating forward- and back-projection and the time is significant (>1 s). On the other hand, our proposed approach involves analytical reconstruction followed by the restoration of noisy projections that do not involve such heavy matrix operations. Note that GPU implementation and the parallel processing of TVNLM is currently an active research area and was beyond the scope of our study. However, a recent report has shown that reconstruction times of 30 ms/image are achievable when the process is parallelized appropriately (18). In future studies, we will implement TVNLM using a GPU and test the acceleration factor compared to state-of-art GPU-based reconstruction frameworks.

**Figure 8:**
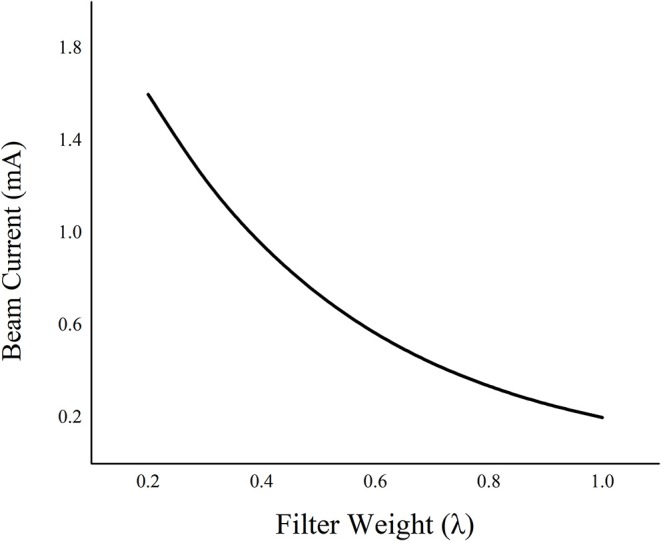
A trade-off relationship between the beam current and filter weight.

One of the main challenges in optimizing the workflow of the proposed framework is choosing an optimal filter weight λ (Equation 1). λ needs to be carefully selected by considering the experimental parameters such as the x-ray current (mA) or the number of projections, and the characteristics of the anatomical features of the scanning object. During the search to find an optimal parameter for λ, we experienced contrast-noise tradeoff. In other words, when the value of λ becomes larger, more penalties are given to the noise reduction term resulting in blurrier projections. In constrast, when the value of λ becomes too small, less penalties are given to the noise reduction term resulting in noisier projections. During the experiments, we studied that a higher λ is practical for lower mA conditions to reduce the noise generated from the photon starvation effect, whereas a lower λ is desirable for higher mA conditions to avoid blurring the anatomical detail of the object. In our study, the empirical choice of λ for reconstructing the numerical phantom with additive Gaussian noise of 0.2 was 0.2, while λ = 1 was used to reconstruct projections of the physical phantom with 1.6 mA, which well reflects the theoretical expectation. As shown in Figure 8, as the λ increases when the current is reduced; therefore, the noise needs to be suppressed by increasing the regularization weight. Nonetheless, further studies are necessary to automatically and robustly estimate λ, and we anticipate that such automation will enhance the performance of our algorithm to become a clinically practical solution. Beam hardening related to Figure 7 is the other pre-processing step, so it was beyond the scope of this study. However, further work will be performed to evaluate the clinical significance of this methodology on developing an optimal reconstruction algorithm adding both beam hardening and scatter correction.

## Conclusion

In this study, we propose a fast and efficient low-dose CBCT reconstruction method by restoring low-dose CBCT projection data with the non-local total variation method. Validation studies showed that the proposed method adds great potential value to enable low-dose CBCT while maintaining image quality acceptable for on-board target localization and delineation. With more extensive validation, we anticipate that our proposed NLTV-GPSR method can be applied to clinical settings enabling significant dose reduction compared to a current clinical protocol.

## Data Availability Statement

All datasets generated for this study are included in the article/supplementary files.

## Author Contributions

JS mainly simulated and wrote the manuscript. CK supervised this study. DK supported the software used. S-RL analyzed some of the results. JZ proofread and corrected the flow of the manuscript. XY modified the equations and put an idea in the discussion part. TL managed all related study and supported.

### Conflict of Interest

The authors declare that the research was conducted in the absence of any commercial or financial relationships that could be construed as a potential conflict of interest.
